# Supplementary material for: Mitigating oxygen loss to improve the cycling performance of high capacity cation-disordered cathode materials
Source: Nat Commun. 2017 Oct 17;8:981. doi: 10.1038/s41467-017-01115-0 (PMC5645360; doi:10.1038/s41467-017-01115-0)
Supplement: Supplementary file 1 — Supplementary Information [file 41467_2017_1115_MOESM1_ESM.pdf]

Supplementary Information

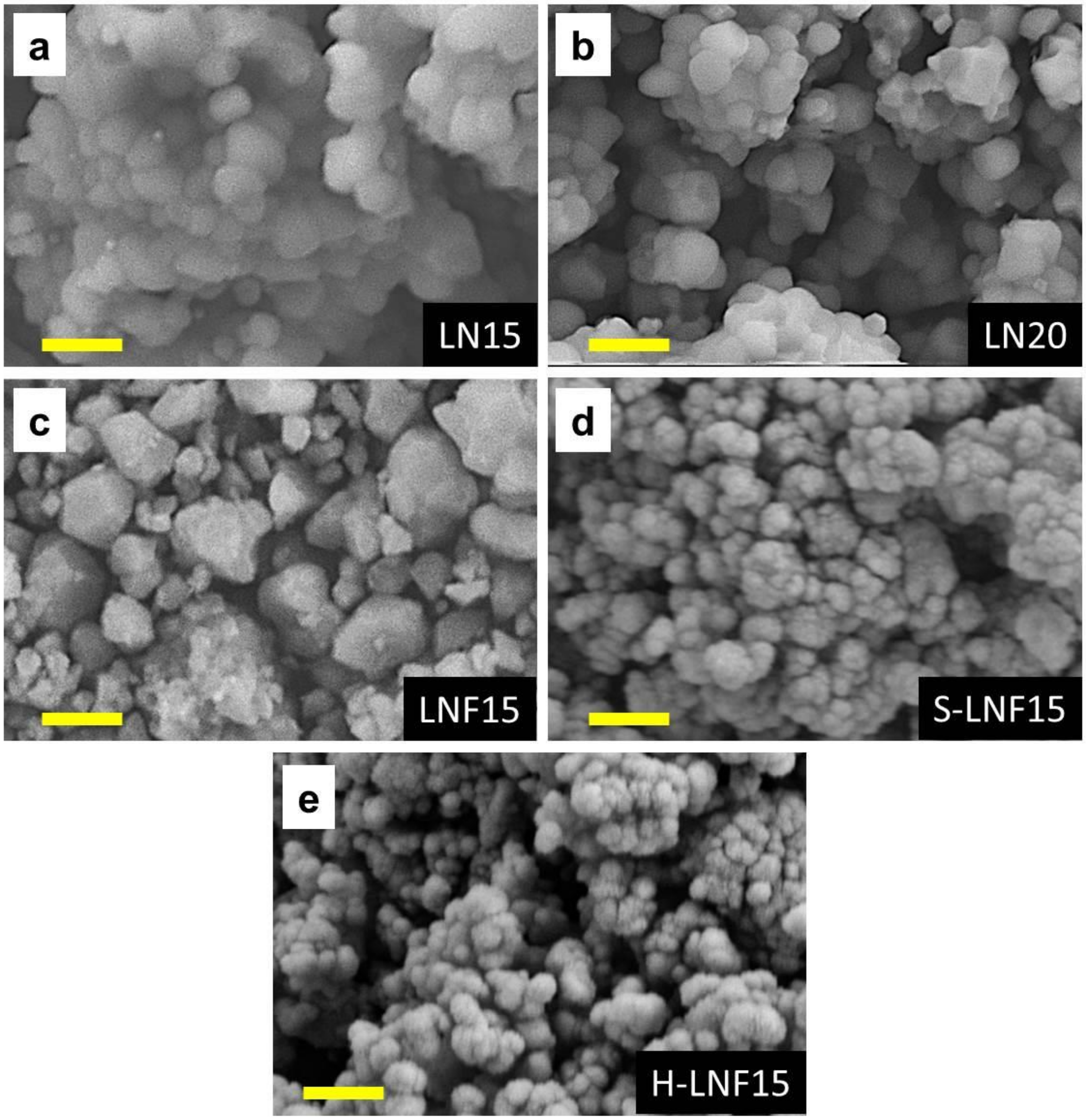

Supplementary Figure 1 Particle morphology of powder samples. The scanning electron microscopy images of **a** $Li_{1.15}Ni_{0.375}Ti_{0.375}Mo_{0.1}O_2$ (LN15), **b** $Li_{1.2}Ni_{0.333}Ti_{0.333}Mo_{0.133}O_2$ (LN20), **c** $Li_{1.15}Ni_{0.45}Ti_{0.3}Mo_{0.1}O_{1.85}F_{0.15}$ (LNF15), **d** shaker-milled LNF15 (S-LNF15), and **e** high-energy ball milled LNF15 (H-LNF15). Scale bars, 200 nm.

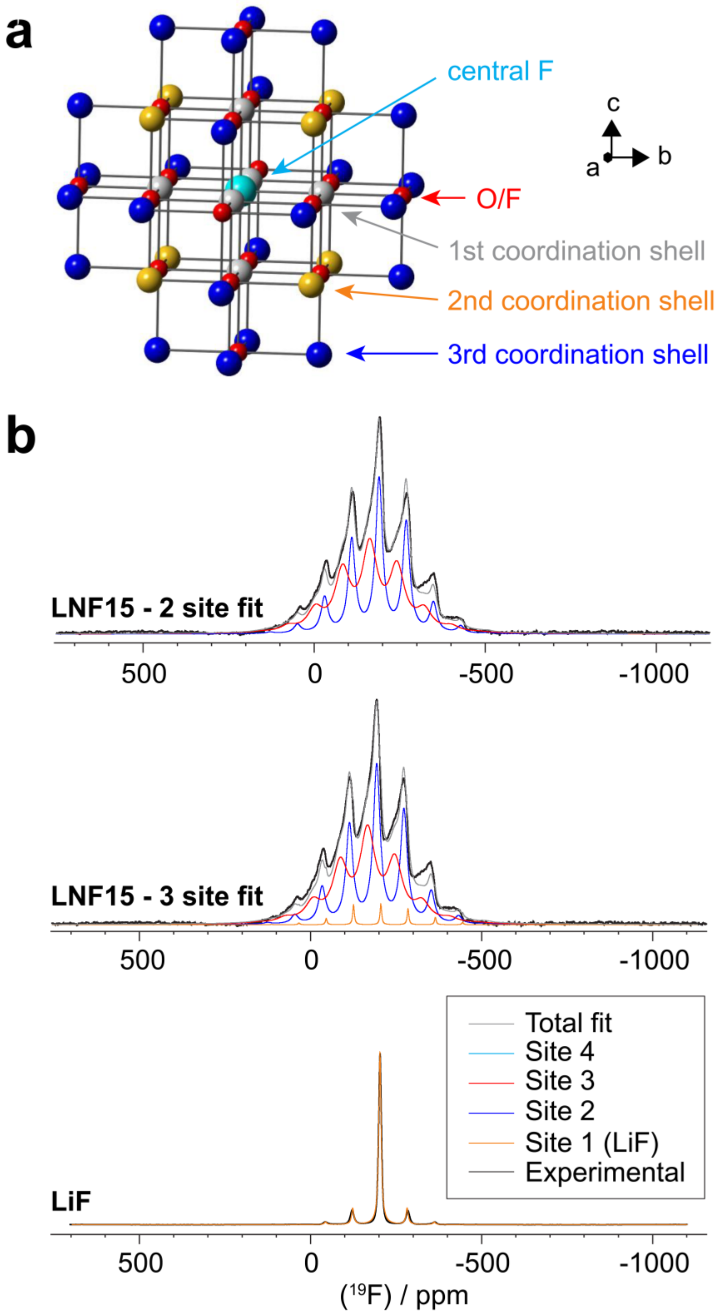

Supplementary Figure 2 Analysis of ^{19}F spin echo NMR (nuclear magnetic resonance) spectra **a** Fluorine local environment, showing the first three metal (transition metal or Li) coordination shells within a radius of 5 Å from the central F atom. **b** Fitted ^{19}F NMR spectra collected on $Li_{1.15}Ni_{0.45}Ti_{0.3}Mo_{0.1}O_{1.85}F_{0.15}$ (LNF15) and LiF. The spin echo spectrum obtained for LiF was fitted with a single Li site, while that for LNF15 was fitted using two and three different F sites. The fitting parameters are listed in Supplementary Table 2.

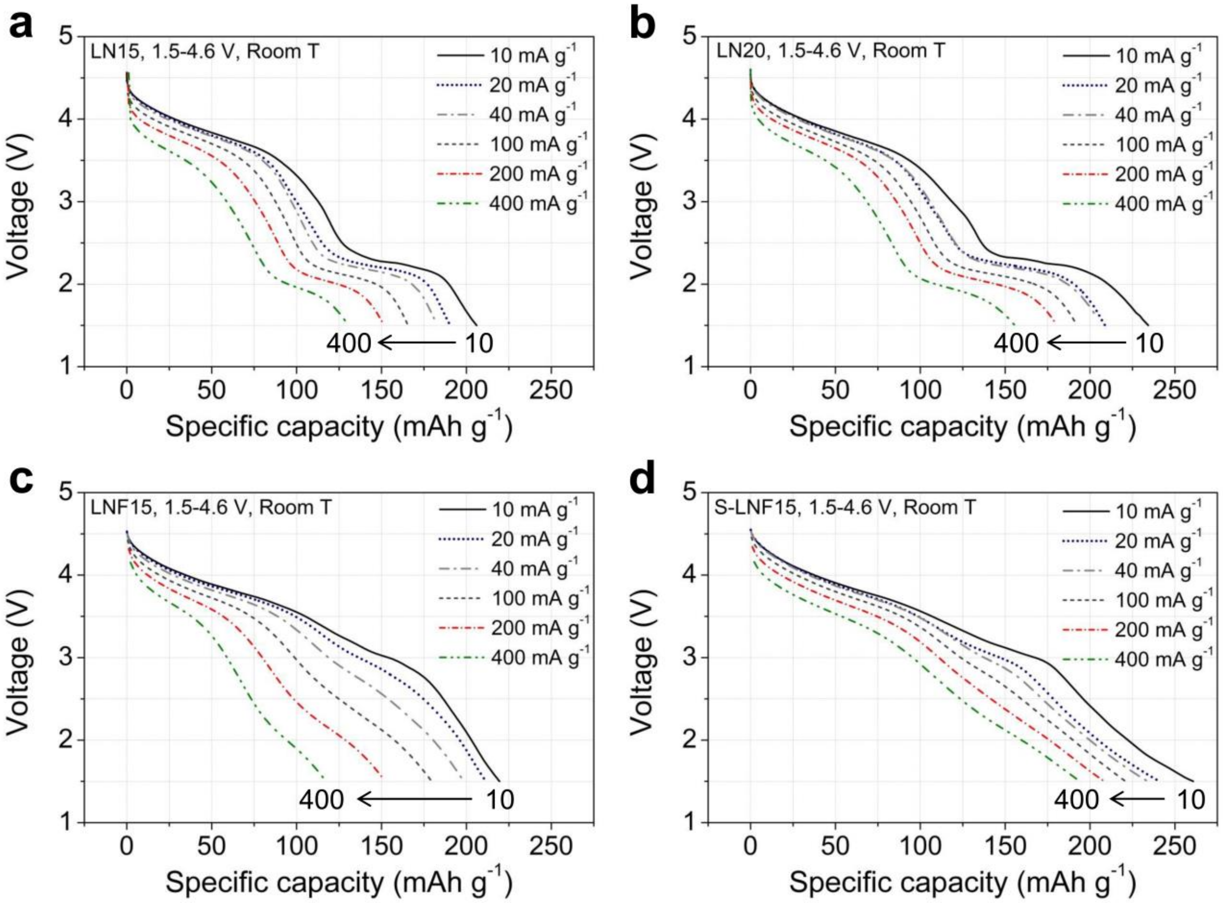

Supplementary Figure 3 Rate capability tests. Discharge voltage profiles of **a** $Li_{1.15}Ni_{0.375}Ti_{0.375}Mo_{0.1}O_2$ (LN15), **b** $Li_{1.2}Ni_{0.333}Ti_{0.333}Mo_{0.133}O_2$ (LN20), **c** $Li_{1.15}Ni_{0.45}Ti_{0.3}Mo_{0.1}O_{1.85}F_{0.15}$ (LNF15), and **d** shaker milled LNF15 (S-LNF15), when charged at a rate of 20 mA g^{-1} and discharged at different rates (10, 20, 40, 100, 200 and 400 mA g^{-1}) between 1.5 and 4.6 V at room temperature. As the discharge rate increases from 10 to 400 mA g^{-1}, the discharge capacity decreases from 206 to 130 mAh g^{-1} for LN15, 234 to 155 mAh g^{-1} for LN20, 214 to 117 mAh g^{-1} for LNF15, and 261 to 194 mAh g^{-1} for S-LNF15. Due to the different microstructures of LN15, LN20 and (S-)LNF15, a direct comparison of the rate capabilities may not give a fair account of the relative performance of the various compounds. For instance, the particle size of as-synthesized LNF15 powder is larger than that of LN15 and LN20 (Supplementary Fig. 1). Thus, while the rate capability of as synthesized LNF15 seems poorer than LN20 at first glance, its rate capability becomes better when the particle size is reduced by shaker milling (S-LNF15).

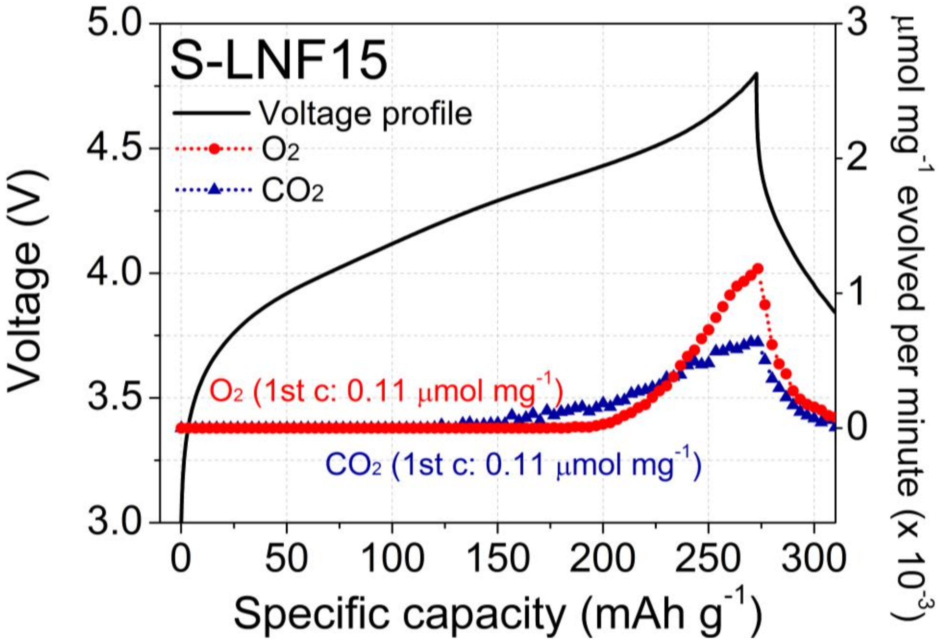

Supplementary Figure 4 Differential electrochemical mass-spectrometry (DEMS) study of shaker milled $Li_{1.15}Ni_{0.45}Ti_{0.3}Mo_{0.1}O_{1.85}F_{0.15}$ (S-LNF15). Voltage profiles (black solid) of S-LNF15, when charged to 4.8 V and discharged to 1.5 V at a rate of 20 mA g^{-1}. The DEMS results for O_2 [red circle] and CO_2 [blue triangle] evolution are also shown.

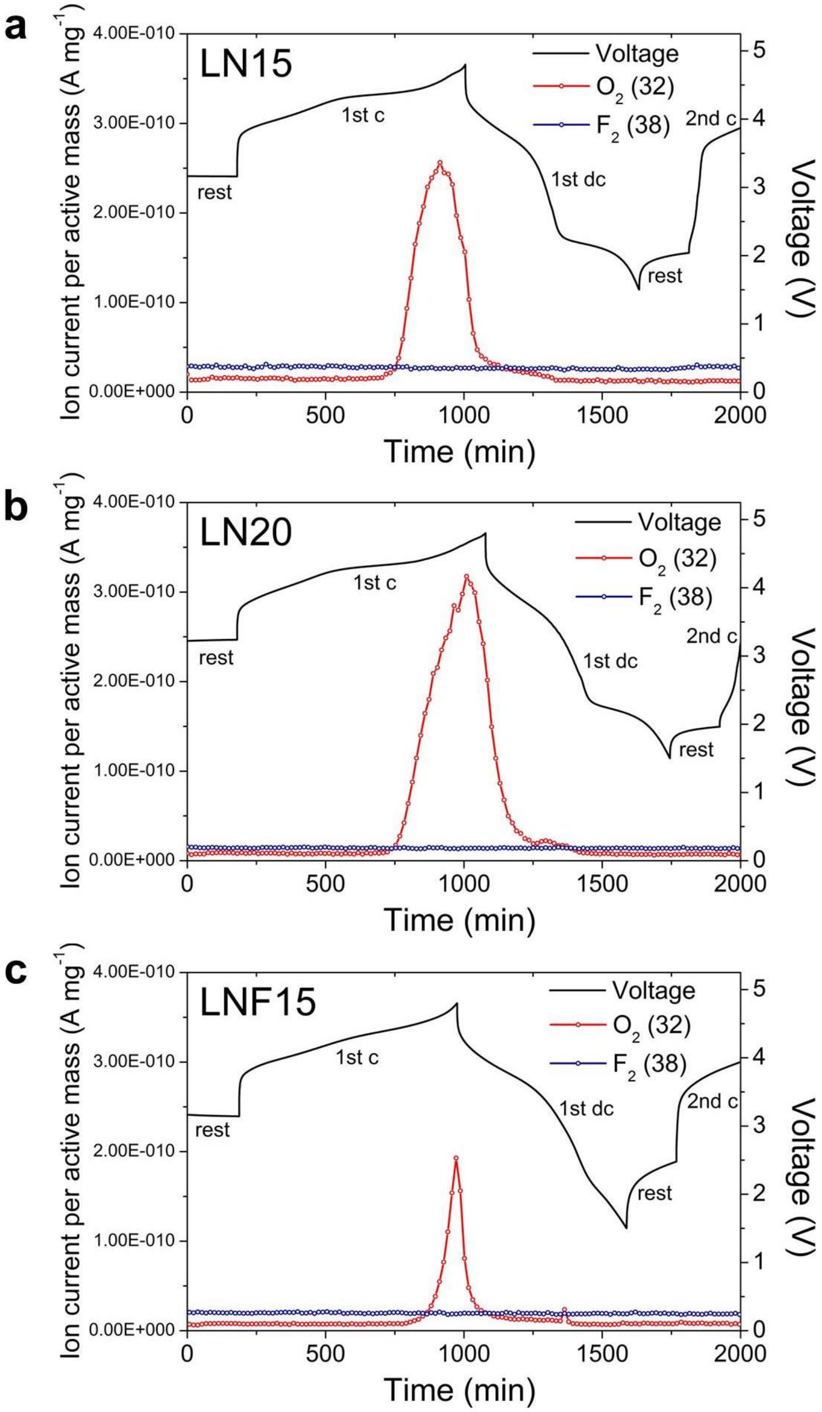

Supplementary Figure 5 Differential electrochemical mass-spectrometry (DEMS) study. With the voltage profiles, we show the ion current signals from O_2 (g) and F_2 (g) evolution, per mg (A mg^{-1}) of **a** $Li_{1.15}Ni_{0.375}Ti_{0.375}Mo_{0.1}O_2$ (LN15), **b** $Li_{1.2}Ni_{0.333}Ti_{0.333}Mo_{0.133}O_2$ (LN20), and **c** $Li_{1.15}Ni_{0.45}Ti_{0.3}Mo_{0.1}O_{1.85}F_{0.15}$ (LNF15), when they are charged to 4.8 V and discharged to 1.5 V at a rate of 20 mA g^{-1}. F_2 signal for LNF15 is silent as is the case for LN15 and LN20, indicating no F_2 (g) evolution from LNF15.

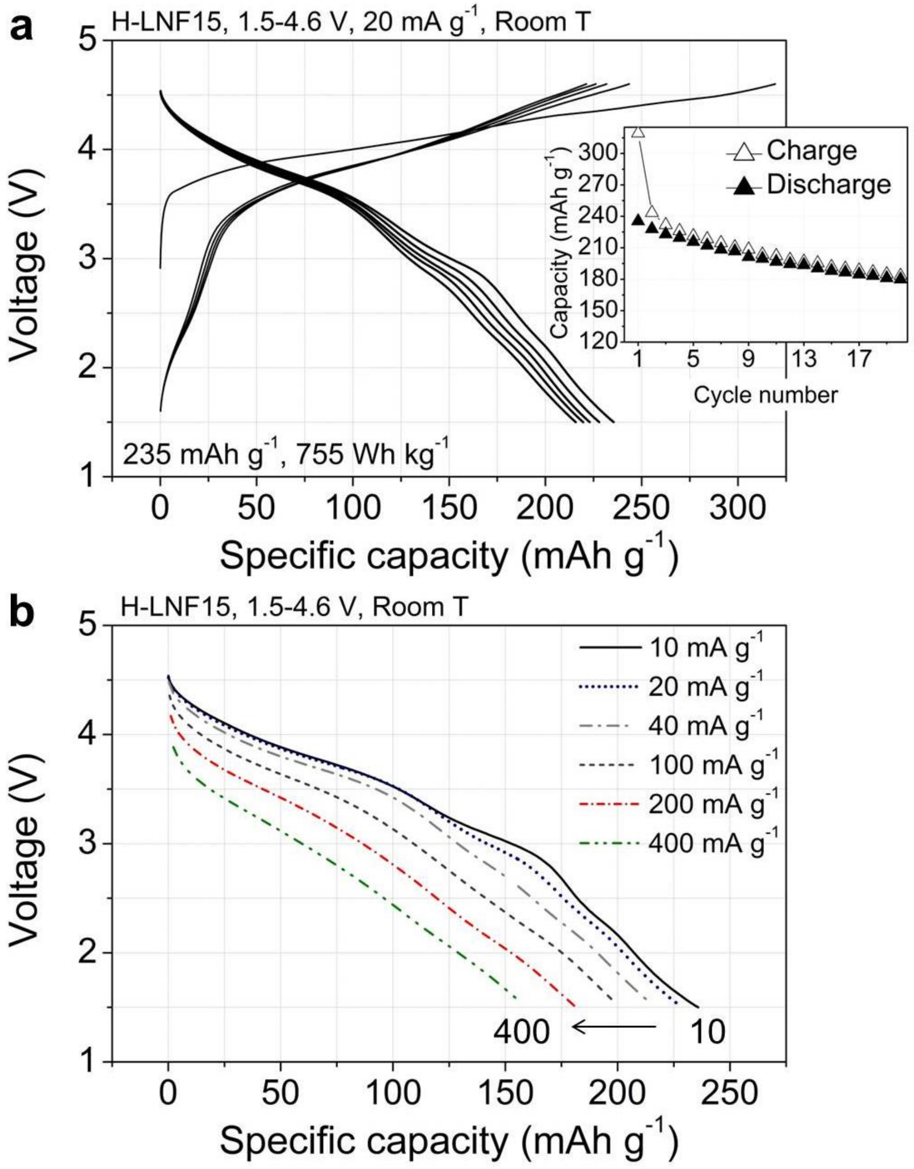

Supplementary Figure 6 Electrochemical properties of high-energy ball milled $Li_{1.15}Ni_{0.45}Ti_{0.3}Mo_{0.1}O_{1.85}F_{0.15}$ (H-LNF15) **a** Voltage profiles of H-LNF15, when cycled between 1.5–4.6 V at a rate of 20 mA g^{-1} at room temperature. The inset shows the capacity retention of the materials over the first 20 cycles. **b** The discharge voltage profiles of H-LNF15, when charged at 20 mA g^{-1} and discharged at different rates of 10, 20, 40, 100, 200 and 400 mA g^{-1} between 1.5 and 4.6 V at room temperature.

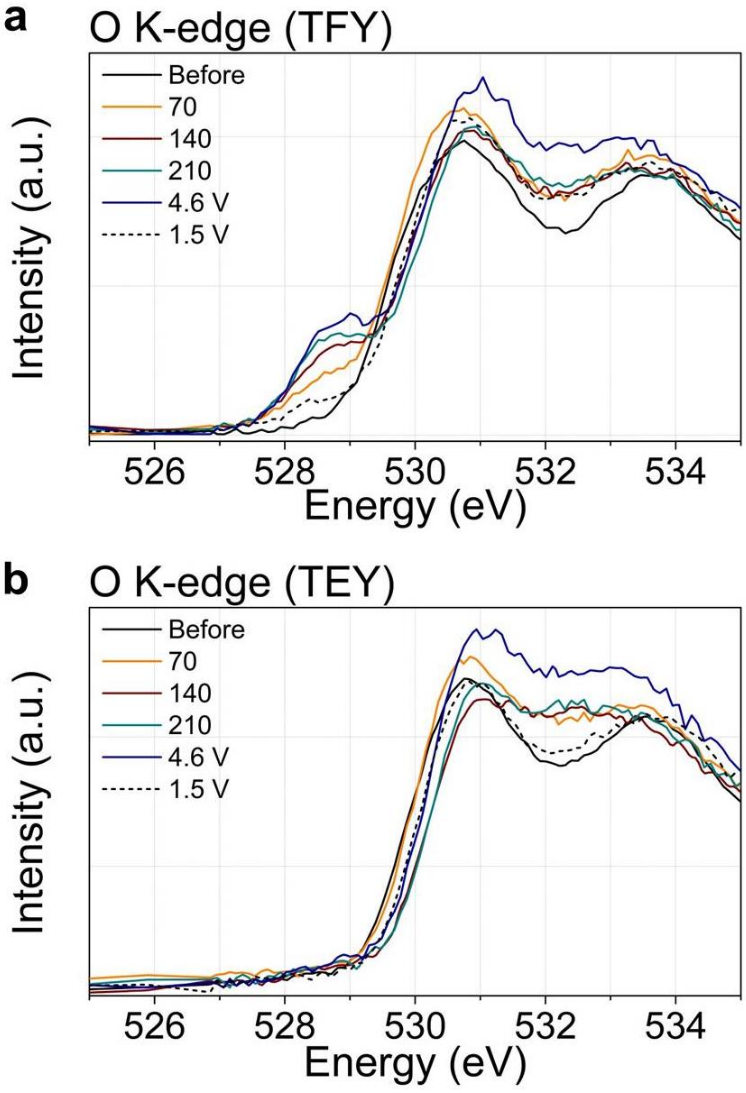

Supplementary Figure 7 Soft X-ray absorption spectroscopy (sXAS) study of high-energy ball milled $Li_{1.15}Ni_{0.45}Ti_{0.3}Mo_{0.1}O_{1.85}F_{0.15}$ (H-LNF15). sXAS spectra at the O K-edge of H-LNF15: **a** total fluorescence yield (TFY) mode, **b** total electron yield (TEY) mode. The data was collected before cycling H-LNF15 (black), after charging to 70 mAh g^{-1} (orange), 140 mAh g^{-1} (dark red), 210 mAh g^{-1} (turquoise), 4.6 V (~280 mAh g^{-1}, dark blue), and after discharging to 1.5 V (black dash). H-LNF15 was cycled at 20 mA g^{-1} at room temperature.

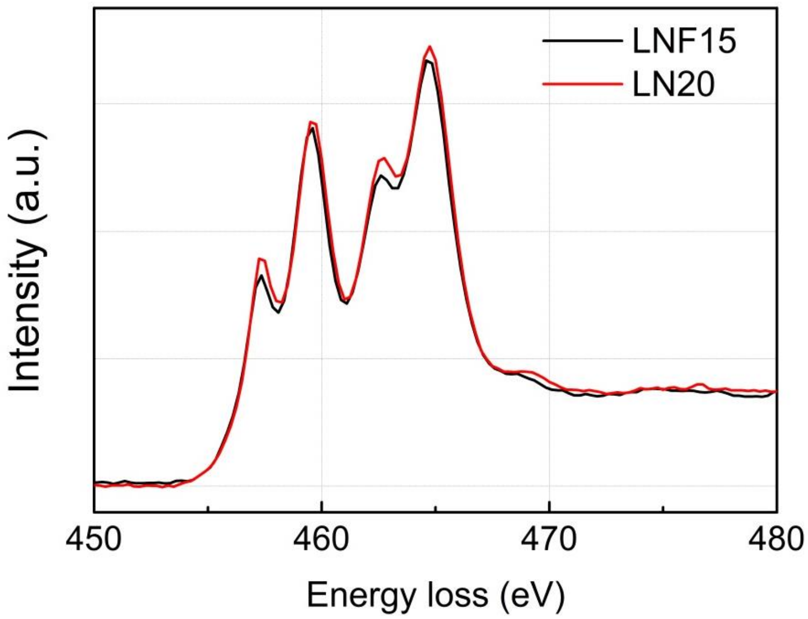

Supplementary Figure 8 Electron energy loss spectroscopy (EELS) study of $Li_{1.2}Ni_{0.333}Ti_{0.333}Mo_{0.133}O_2$ (LN20) and $Li_{1.15}Ni_{0.45}Ti_{0.3}Mo_{0.1}O_{1.85}F_{0.15}$ (LNF15). Ti L-edges in as-prepared LNF15 (black) and LN20 (red) are shown. The two spectra are very similar to each other, indicating that Ti is in the Ti^{4+} state in LNF15 as in LN20.

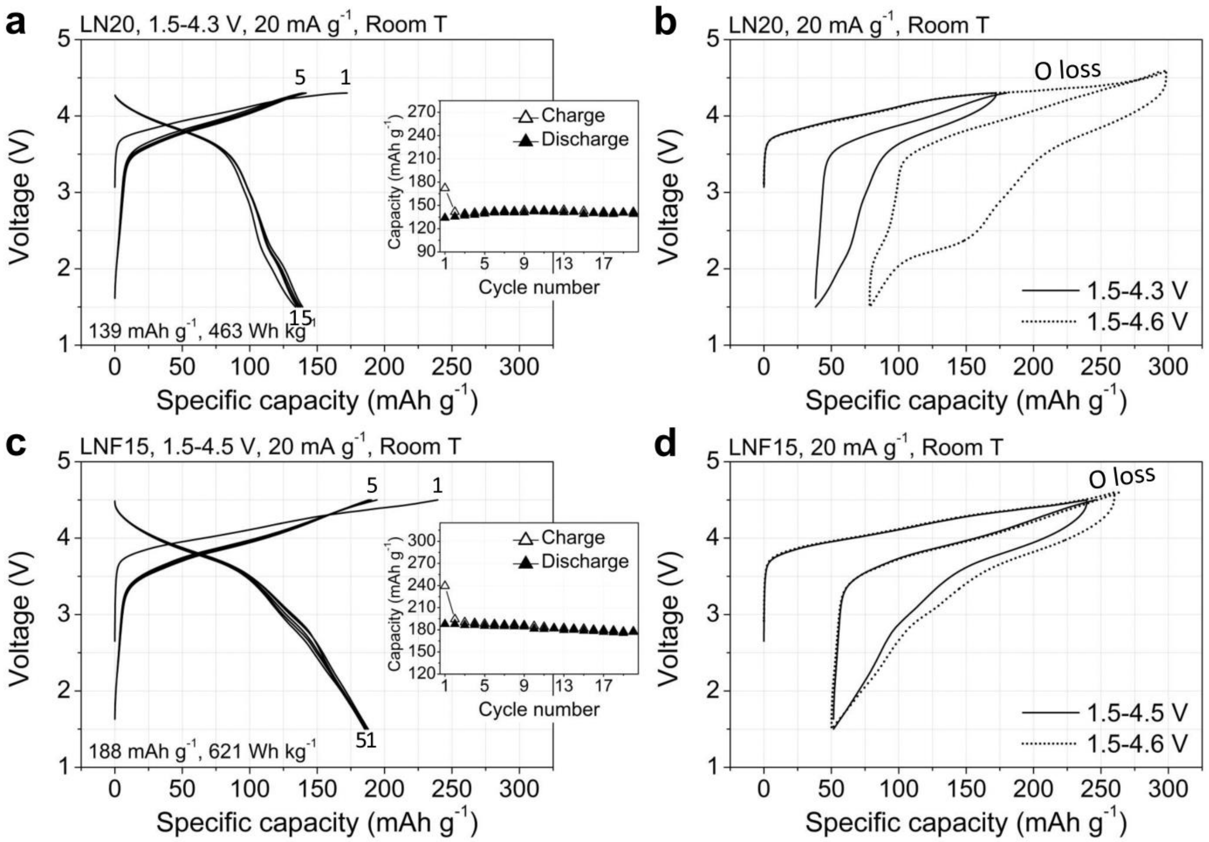

Supplementary Figure 9 Effects of voltage window to the cycling performance of $Li_{1.2}Ni_{0.333}Ti_{0.333}Mo_{0.133}O_2$ (LN20) and $Li_{1.15}Ni_{0.45}Ti_{0.3}Mo_{0.1}O_{1.85}F_{0.15}$ (LNF15) **a** Voltage profiles and capacity retention of LN20, when cycled between 1.5–4.3 V at a rate of 20 mA g^{-1} at room temperature. **b** Voltage profiles of LN20 obtained upon first-cycle and second-charge between 1.5–4.3 V (solid) and between 1.5–4.6 V (dot). LN20 exhibits large polarization when charged to 4.5 V, but it shows much reduced polarization if charging is limited to 4.3 V, which is below the oxygen loss threshold of LN20. This result demonstrates how oxygen loss via cation densification affects the performance of cation-disordered materials. **c** Voltage profiles and capacity retention of LNF15, when cycled between 1.5–4.5 V at a rate of 20 mA g^{-1} at room temperature. **d** Voltage profiles of LNF15 obtained upon first-cycle and second-charge between 1.5–4.5 V (solid) and between 1.5–4.6 V (dot). LNF15 also exhibits slightly reduced polarization if charging is limited to below its oxygen loss threshold (4.5 V).

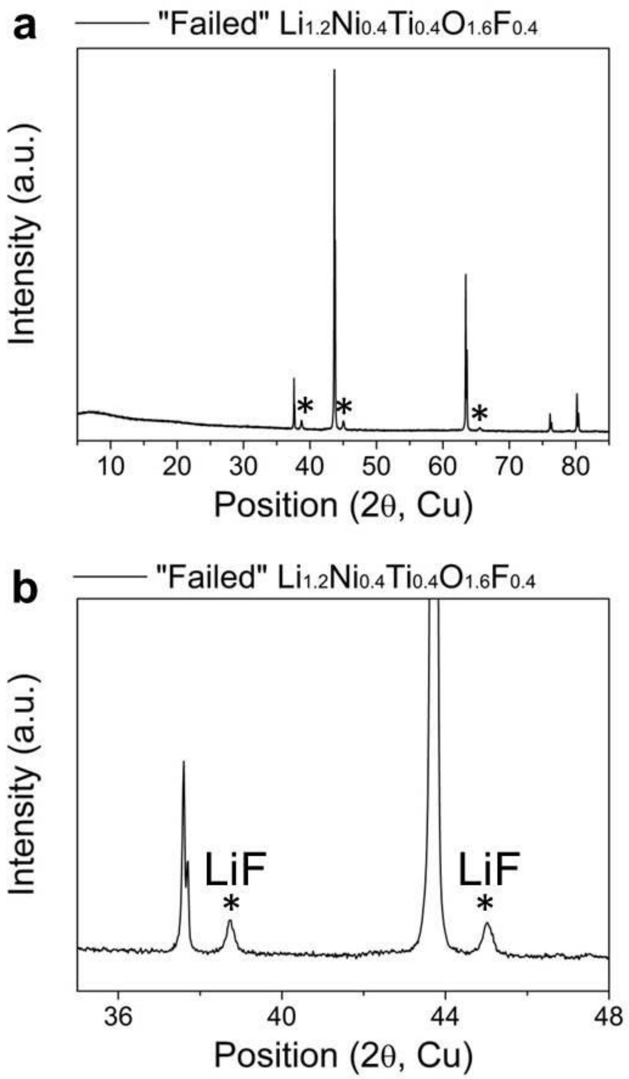

Supplementary Figure 10 An example of solubility limits of fluorine to Li-excess cation-disordered rocksalt oxides. **a** X-ray diffraction (XRD) pattern obtained in an attempt to synthesize $Li_{1.2}Ni_{0.4}Ti_{0.4}O_{1.6}F_{0.4}$ (900°C, 5 hours, air). **b** An enlargement of the XRD pattern showing the 2Θ range from 35 to 48°. LiF peaks are indicated with (*).

Supplementary Table 1 Structural parameters from X-ray diffraction (XRD) Rietveld refinements: LN15 = $Li_{1.15}Ni_{0.375}Ti_{0.375}Mo_{0.1}O_2$, LN20 = $Li_{1.2}Ni_{0.333}Ti_{0.333}Mo_{0.133}O_2$, LNF15 = $Li_{1.15}Ni_{0.45}Ti_{0.3}Mo_{0.1}O_{1.85}F_{0.15}$. Crystallographic information file of Fm-3m $LiFeO_2$ (ICSD collection code 51208) was used as an input file. Pseudo-Voigt fit was used: (U, V, W) = (-0.017289, 0.109226, 0.013451) for LN15, (0.094804, -0.091479, 0.054049) for LN20, and (0.165450, -0.084446, 0.051998) for LNF15. $B_{isotropic}$ factor was fixed to 0.5 $Å^2$ for all elements. The atomic occupancies were initially set to the atomic ratio obtained from elemental analysis by direct current plasma emission spectroscopy and an ion selective electrode, based on which the lattice parameters were first refined. Then, we further refined the lattice parameters and the atomic occupancies simultaneously: transition metal (TM) occupancies were first refined, and then Li occupancy was refined. O and F occupancies of LNF15 were refined after the TM and Li occupancies were refined and then fixed, but their values barely changed from their initial values set based on elemental analysis. Nevertheless, since O and F are hardly distinguishable by XRD, the refined O and F occupancies are more subject to error. Hence, we performed solid state NMR to prove the fluorine substitution in the bulk lattice of LNF15 (Supplementary Fig. 2, Supplementary Table 2, and Supplementary Note 1). O occupancy did not change after the refinement for LN15 and LN20.

**Material**		**LN15**	**LN20**	**LNF15**
Space group			Fm-3m	
R_{wp} / Goodness of fit		2.903 / 7.006	2.095 / 5.493	2.913 / 4.690
Site 4a (x, y, z) = (0, 0, 0)	Li occupancy	0.5680	0.5894	0.5659
	Ni occupancy	0.1885	0.1694	0.2216
	Ti occupancy	0.1845	0.1661	0.1500
	Mo occupancy	0.0515	0.0686	0.0503
Site 4b (x, y, z) = (0.5, 0.5, 0.5)	O occupancy	1	1	0.9292
	F occupancy	0	0	0.0710
a (Å)		4.14444	4.14486	4.14146
Volume ($Å^3$)		71.1968	71.20815	71.0328
Derived density (kg l^{-1})		4.18	4.16	4.22

Supplementary Table 2 Nuclear magnetic resonance (NMR) parameters obtained from the fits of the ^{19}F spin echo NMR spectra presented in Figure 3 in the main paper. In this Supplementary Table 2, the isotropic shift (δ_{iso}, in ppm), dipolar anisotropy ($\Delta_\delta = \delta_{ZZ} - \frac{1}{2}(\delta_{XX} + \delta_{YY})$, in ppm), dipolar asymmetry (η), line broadening (LB, in kHz), percentage of the total NMR signal integrated intensity (% integr. intensity), and goodness of fit (gof, in %) are recorded. LNF15 = $Li_{1.15}Ni_{0.45}Ti_{0.3}Mo_{0.1}O_{1.85}F_{0.15}$.

Compound/Fit	Compound/Fit	Site 1	Site 2	Site 3
**LiF (gof = 87.0 %)**	δ_{iso}/ppm	-203	–	–
	Δ_δ/ppm	80	–	–
	η	1.0	–	–
	LB / kHz	3.1	–	–
	% integr. intensity	100.0	–	–
**LNF15 – 3 site fit (gof = 93.7 %)**	δ_{iso}/ppm	-203	-193	-166
	Δ_δ/ppm	-217	192	198
	η	0.8	0.9	0.9
	LB / kHz	2.2	9.0	20.1
	% integr. intensity	1.7	42.3	56.0
**LNF15 – 2 site fit (gof = 93.6 %)**	δ_{iso}/ppm	–	-194	-167
	Δ_δ/ppm	–	194	196
	η	–	0.8	0.9
	LB / kHz	–	9.1	20.3
	% integr. intensity	–	44.0	56.0

Supplementary Note 1

To gain insight into the various fluorine local environments present in as-synthesized $Li_{1.15}Ni_{0.45}Ti_{0.3}Mo_{0.1}O_{1.85}F_{0.15}$ (LNF15), NMR parameters were determined from fits of the experimental ^{19}F NMR data (Supplementary Fig. 2b). A fit of the ^{19}F spectrum collected on LiF yielded NMR parameters for the unique fluorine site in the material, denoted as site 1 (Supplementary Fig. 2b and Supplementary Table 2). All fitted NMR parameters are recorded in the Supplementary Table 2.

Since little is known about the different F sites present in LNF15, two different fits were performed, considering two and three different F environments, respectively. Nevertheless, the two fits are closely related, consisting of two major F environments, denoted as sites 2 and 3 (Supplementary Fig. 2b and Supplementary Table 2). An additional F environment was considered in the three-site fit to quantify the contribution from a F site closely related to the unique site observed in LiF (site 1). In fact, the chemical shift of site 1 in the three-site fit was fixed to that obtained for the unique F site in LiF. The results of the three-site fit indicate that LiF-like F environments, if present, only account for a very small proportion of the total F content in the material. This result suggests that, if a secondary LiF phase is formed (*e.g.* at the surface of LNF15 particles), it is present as a minor phase. The significantly broader line shape of site 1 in the three-site fit of the LNF15 spectrum, as compared with site 1 in the fit of the LiF spectrum, may be accounted for by the presence of additional interactions between the ^{19}F nucleus and nearby unpaired electrons (*i.e.*, paramagnetic interactions) in the fluorinated material. These observations are consistent with the formation of a thin LiF-like layer at the surface of the cathode particles, as was previously reported for $Li_{1.1}(Ni_{0.425}Mn_{0.425}Co_{0.15})_{0.9}O_{1.8}F_{0.2}$.[1,2]

On the other hand, the NMR parameters obtained for site 2 and site 3 are very different from those obtained for the unique F site in LiF. The shifts of the resonant frequencies, of ~ -193 ppm (site 2) and ~ -164 ppm (site 3), respectively, as compared with -203 ppm for the single F site in LiF, as well as the large $\Delta\delta$ values and broad lines, of 192 ppm and 9.0 kHz for site 2, and of 198 ppm and 20.1 kHz for site 3 (parameters obtained from the three-site fit), presumably arise from paramagnetic interactions between the F nuclei and unpaired electrons from nearby Ni^{2+} cations. Paramagnetic interactions are relatively short-range, suggesting that F and Ni species are less than 5 Å, i.e. that F is incorporated in the bulk lattice. In addition, the presence of multiple F

resonances with different isotropic shifts in the spectrum of LNF15 is consistent with the presence of at least two different F environments in the material, with different TM configurations in the first and second TM coordination shells (Supplementary Fig. 2a). Site 2, with a shift fairly close to that of the unique F site in LiF, is tentatively assigned to a F site with 6 Li in its first metal coordination shell, and paramagnetic Ni^{2+} ions further away from the central F atom. Site 3, on the other hand, may have one (or more) Ni ion(s) in the first, second or third metal coordination shell, leading to a less negative shift and to a broader resonance.

Supplementary References

1. Ménétrier, M. *et al.*, NMR evidence of LiF coating rather than fluorine substitution in $Li(Ni_{0.425}Mn_{0.425}Co_{0.15})O_2$. *J. Solid State Chem.* **181**, 3303–3307 (2008).
2. Croguennec, L. *et al.*, Synthesis of "$Li_{1.1}(Ni_{0.425}Mn_{0.425}Co_{0.15})_{0.9}O_{1.8}F_{0.2}$" Materials by Different Routes: Is There Fluorine Substitution for Oxygen? *J. Electrochem. Soc.*, **156**, A349–A355 (2009).
